# The Great Pyramids

**Published:** 1874-03

**Authors:** 


### THE GREAT PYRAMIDS.

It was 300 years B. C. when the Greeks commenced their proverb about the seven wonders of the world, and named the pyramids first. It (the largest of them) has four sides, which face the four cardinal points of the compass with more exactness than can be determined by the compass itself without the aid of calculation. It is built of dressed and systematically adjusted limestone, and some of the stones were carried from quarries 500 miles distant. It consists of 70,000,000 cubic feet of masonry. It covers more than twelve acres. It is a perfect square at its base, has four equal sides, and has but one narrow passage, which pierces it on the north side directly on the meridian. The opening runs at an angle pointing outward to the then polar star. It was built 2170 B. C., and only then the Pleiades and the then polar star were, at midnight, in October, exactly opposite each other, and both were on the meridian. Sir John Herschel, thirty years ago, thus fixed upon the date of the pyramid as embodied unmistakably in itself. The same configuration of the heavens cannot recur, from that time, for 25,868 years. At the rate of one inch for a year the number of years in the whole processional cycle is built in the sum of the two diagonals of the base. Its height is to twice its breadth as the diameter to the circumference of the circle, and thus it stands in its whole shape a type and memorial of the squaring of the circle, performed ages and ages before the question was ever heard of among the schools of philosophy or the written records of mathematical investigation. A hebdomadal system also appears in this greatest of human structures. The astronomical intelligence embodied in this great pyramid is equally wonderful. It is not only oriented, as above stated, but each side of its base measures 365 cubits, the number of days in the year, with a slight addition in each, which together make up for the nearly six hours additional which, in the four years, require one day to be added, as in "leap year." The height of the pyramid multiplied by 10 to the 9th power gives the distance from the earth to the sun, almost precisely as most recently calculated in miles, and most probably with greater accuracy than our modern science, which still labors under some uncertainty in regard to this point. Its chief chamber is so arranged as to give the mean temperature of the whole surface of the habitable globe 68° Fahrenheit. Many other wonderful things are recorded about it, which cannot be mere coincidences, but are unmistakable evidences of real scientific knowledge.

